# Supplementary material for: How big five personality traits influence information sharing on social media: A meta analysis
Source: PLoS One. 2024 Jun 12;19(6):e0303770. doi: 10.1371/journal.pone.0303770 (PMC11168692; doi:10.1371/journal.pone.0303770)
Supplement: Attachment — Submitted filename: Response to Reviewers.pdf [file pone.0303770.s003.pdf]

We appreciate all reviewers for recognizing the novelty of our paper. Below, we provide the point-by-point responses for each reviewer.

Comment 1: In particular, we note that there is no assessment of the quality or risk of bias of the included studies. Conclusions in a systematic review or meta-analysis should be related to the quality of the included publications. Without this critical assessment of the included studies, we do not feel that your systematic review currently fulfils our requirements for consideration (for more information, we would refer you to the following source: Higgins JPT, Thomas J, Chandler J, Cumpston M, Li T, Page MJ, Welch VA (editors).

Reply 1: We have added sections 4.5 Quality Assessment in revised manuscript. We adopted quality assessment tools that are being used in meta-analysis in the area of Engineering.

4.5 Quality Assessment

Although most quality checklists published in extant academic literature have primarily addressed medical studies, we sought to ensure the thorough evaluation of the selected studies by adhering to a combination of established guidelines. To rigorously assess the methodological quality of the studies included in this meta-analysis, we followed the guidelines of Kitchenham and Charters [46] as well as the meta-analysis on the existing quality assessment tools that are being used in meta-analysis in the area of

Engineering [47]. The study suggested using a set of questions based on widely used checklists and guidelines for the design, conduct, analysis, and conclusions of each study in this meta-analysis. The study evaluation criteria were based on the questions presented below.

- **Q1**: Are the aims of the research clearly defined?
- **Q2**: Is there an adequate description of the context in which the research was carried out?
- **Q3**: Was the research design appropriate to address the aims of the research?
- **Q4**: Was there a control group?
- **Q5**: Are the data collection methods adequately described?
- **Q6**: Were all measures used in the study fully defined?
- **Q7**: Is the experimental design appropriate and justifiable?
- **Q8**: Does the study provide description and justification of the data analysis approaches?
- **Q9**: Are the findings of the study clearly stated?
- **Q10**: Does the study add value to academia or practice?

The scoring procedure assigned a value of 1 for ”Yes” and 0 for ”No”. Studies could score between 0 and 10 points. Papers receiving a score exceeding 8 (¿8) were decided to be retained in this meta-analysis. The results of quality assessment are presented in S2 Table.

Table 4. Result of quality assessment

Study	Title	Score
David et al. 2012	A tale of two sites: Twitter vs. Facebook and the personality predictors of social media usage	9
Chen 2016	The Influences of Personality and Motivation on the Sharing of Misinformation on Social Media	9
Homero et al. 2017	Personality Traits and Social Media Use in 20 Countries : How Personality Relates to Frequency of Social Media Use Social Media News Use, and Social Media Use for Social Interaction	10
Deng et al. 2017	How do personality traits shape information-sharing behaviour in social media? Exploring the mediating effect of generalized trust	9
Liu et al. 2017	社会化商务下个体心理因素对信息共享行为的影响研究——大五人格的调节作用	9
Mohammad et al. 2018	Sharing Political Content in Online Social Media: A Planned and Unplanned Behaviour Approach	10
Buchanan et al. 2019	Spreading Disinformation on Facebook: Do Trust in Message Source, Risk Propensity, or Personality Affect the Organic Reach of "Fake News"?	10
Damien et al. 2019	Willingness to Share Emotion Information on Social Media: Influence of Personality and Social Context	8
Huang et al. 2020	自媒体用户信息共享行为动机分析与实证	9
Tom 2020	Why do people spread false information online? The effects of message and viewer characteristics on self-reported likelihood of sharing social media disinformation	10
Yin et al. 2020	Reposting negative information on microblogs: Do personality traits matter?	9
Xiao et al. 2021	Wired to seek, comment and share? Examining the relationship between personality, news consumption and misinformation engagement	9
Brinda et al. 2022	Fake or real news? Understanding the gratifications and personality traits of individuals sharing fake news on social media platforms	10
Ahmed1 et al. 2022	Social Media News use and covid-19 misinformation engagement: Survey study	9
Xu et al. 2023	EID事件情境下情绪对信息分享行为的动态影响——人格特质的调节作用	8
Kim et al. 2014	Individual Differences in Social Media Use for Information Seeking	6
Luo 2018	社交媒体中用户人格特质对科学信息分享动机的影响与反思	7

Furthermore, we have added some practical implications of our work.

Second, the key research in current information science is personalized applications [55–57], such as recommendation system and chat AI. The results of this study will contribute to the development of these personalized applications. This work

also holds important implications for the field of security, particularly in addressing the prevalent issues of rumor spreading and online fraud. Current social landscape is marred by the substantial impact of these problems. The notion of "psychological persuasion" has gained attention in recent research [58], revealing the potency of personalized warnings in improving the efficacy of persuasion strategies [59, 60]. This study, along with machine learning-based automatic personality detection methods, enables the possibility of delivering personalized warnings on a large scale.
