# Supplementary material for: How big five personality traits influence information sharing on social media: A meta analysis
Source: PLoS One. 2024 Jun 12;19(6):e0303770. doi: 10.1371/journal.pone.0303770 (PMC11168692; doi:10.1371/journal.pone.0303770)
Supplement: Attachment — Submitted filename: Response to Reviewers(2).pdf [file pone.0303770.s004.pdf]

For Reviewer #1:

Q1: It is typical of a Case Study Report, not a Research Article. Therefore, please improve the editing style appropriate for a Research Article.

R1: We thank your suggestion for our writing. We have modified some expressions to make the writing style of the article more clear. For example: “in this study” → “in this meta-analysis”, “this study aimed” → “We aimed”, “All analyses in our work” → “All calculations related to this meta-analysis”.

Q2: please let me know if you used artificial intelligence in your work? If so, to what extent?

R2: We don’t use artificial intelligence in this work.

Q3: In addition, please refer to the impact of experience on personality in the discussion.
I suggest referring to: https://doi.org/10.3389/fpsyg.2022.854804.

R3: We refer this paper in our Introduction.

Q4: After all, verify the abstract and change the keywords to something other than the title.

R4: We thank your suggestion for our keywords. We will modify the keywords to “Personality; Big Five; Information Sharing in social media; meta-analysis; DerSimonian-Laird”.

Reviewer #2:

Q1: Elucidate the trajectory of your research in the introduction, and underscore why this trajectory is of consequence. Despite the current introduction being quite direct and detailing the structure of this paper, it falls short of a specific delineation of the research problem and background.

R1: We thank your suggestion for our writing. We have added some content in the introduction. The following figure shows the research trajectory of the modified introduction.

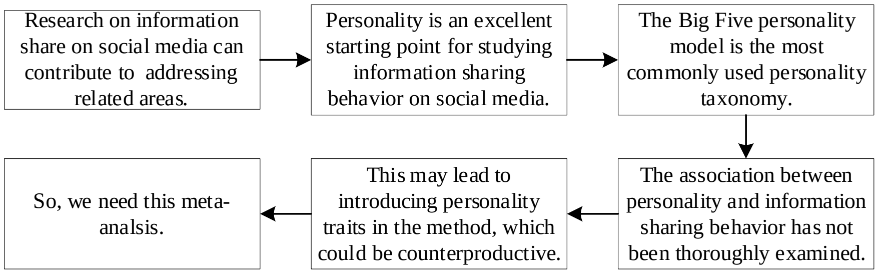

Q2: It is imperative to scrutinise current analogous reviews and accentuate the disparities between this manuscript and them.

R2: We thank your suggestion for our writing. We have added “In addition, there is no published meta-analysis on the relationship between two.” in our introduction.

Q3: The discussion warrants a more profound exploration. Kindly delve deeper into the potential implications of the findings of this article.

R3: We thank your suggestion for our discussion. We have added some content in section “Theoretical and practical implications”.

should incorporate an additional focus on the influence of personality traits. This incorporation will provide a holistic understanding of information sharing behavior. Moreover, studying how personality influences information sharing behavior across different subfields is essential, given the diverse levels of interest that individuals have in

various types of information. For example, individuals with high scores of conscientiousness are more likely to participate in discussions on political related information [56].

Finally, the present study’s findings are particularly noteworthy in light of the current era of artificial intelligence-generated content (e.g., ChatGPT [63]). The fine-tuning of the large language model may also be based on the user’s personality traits. We also recommend personalized strategies when dealing with the dissemination
