# Supplementary material for: How big five personality traits influence information sharing on social media: A meta analysis
Source: PLoS One. 2024 Jun 12;19(6):e0303770. doi: 10.1371/journal.pone.0303770 (PMC11168692; doi:10.1371/journal.pone.0303770)
Supplement: Attachment — Submitted filename: Response to Reviewers2.pdf [file pone.0303770.s005.pdf]

Reviewer #1:

Q1: Since it is now an original article this excessive number of sections and subsections makes the content difficult to perceive. Therefore, consider rebuilding the content breakdown to a few main sections.

R1: We appreciate your acknowledgment of our content, methodology, and pragmatics. We primarily revised Section 6 in accordance with your suggestion, since the standard article structure for Meta analysis was used in the first five Sections. Section 6.2 has been relocated to Section 5 and a discussion on the results has been included.

5.3 Publication bias of subgroup analysis

First, the funnel plot (Fig.9) illustrated that the majority of the studies analyzed are evenly distributed in a symmetrical pattern near the center, suggesting no publication bias in our screened studies by Leave-one-out method.

Futher, table 4 presents the test results of Begg's test and Egger's test, providing the same conclusion as the above. Neither the Begg's test (p=0.19, 0.74,0.788,0.41, 0.65>0.05) nor the Egger's test (p= 0.4347, 0.4161, 0.4372, 0.3632, 0.3020 > 0.05) was significant, providing additional evidence to support the absence of publication bias. This also indicates that there is no publication bias in the studies we selected.

Furthermore, we merged the original sections 6.1, 6.3, and 6.4.

Reviewer #2:

Q1: the selected database is not representative,

R1: We thank your suggestion for our method. I believe you are confused about CNKI as a selected database, as another database, Web of Science, is the most authoritative paper database. CNKI is a highly regarded authoritative database, often selected as the database for many meta-analysis articles published in top journals. For example:

[1] Wang, Shimeng, et al. "Effectiveness of physical activity interventions for core symptoms of autism spectrum disorder: A systematic review and meta-analysis." Autism Research 16.9 (2023): 1811-1824.

[2] Zhang, LiRong, et al. "A meta-analysis of the impact of pharmacist interventions on clinical outcomes in patients with type-2 diabetes." Patient Education and Counseling 120 (2024): 108091.

[3] Bai, Yanping, et al. "Prevalence of Postpartum Depression Based on Diagnostic Interviews: A Systematic Review and Meta-Analysis." Depression and Anxiety 2023 (2023).

[4] Zhu, Maria, et al. "The efficacy of measurement-based care for depressive disorders: systematic review and meta-analysis of randomized controlled trials." The Journal of Clinical Psychiatry 82.5 (2021): 37090.

[5] Du, Xiayu, et al. "Interpretation bias in health anxiety: a systematic review and meta-analysis." Psychological medicine 53.1 (2023): 34-45.

Moreover, we only selected literatures indexed by Core Journals of Peking University. This rule filters out most low-quality articles in CNKI. We have added a discussion on CNKI and references.

> National Knowledge Infrastructure® (CNKI). To ensure the quality of the literature, we only selected literatures indexed by Science Citation Index or Engineering Index (for literature written in English) and Core Journals of Peking University[1] (for literature written in Chinese). This rule filters out most low-quality articles in CNKI [22]. Due to the timeliness of social media research, we only adopt literature from the past decade (2012-2023). Given the main research objectives, we searched literature with multiple [illegible]

Q2: the structure and presentation of the manuscript are not clear enough,

R2: We thank your suggestion for our writing. We primarily revised Section 6 in accordance with your suggestion, since the standard article structure for Meta analysis was used in the first five Sections. Section 6.2 has been relocated to Section 5 and a discussion on the results has been included.

5.3 Publication bias of subgroup analysis

First, the funnel plot (Fig.9) illustrated that the majority of the studies analyzed are evenly distributed in a symmetrical pattern near the center, suggesting no publication bias in our screened studies by Leave-one-out method.

Futher, table 4 presents the test results of Begg's test and Egger's test, providing the same conclusion as the above. Neither the Begg's test (p=0.19, 0.74,0.788,0.41, 0.65>0.05) nor the Egger's test (p= 0.4347, 0.4161, 0.4372, 0.3632, 0.3020 > 0.05) was significant, providing additional evidence to support the absence of publication bias. This also indicates that there is no publication bias in the studies we selected.

Furthermore, we merged the original sections 6.1, 6.3, and 6.4.

In addition, we have once again refined the presentation throughout the entire manuscript.

Q3: the manuscript does not fully discuss the issues of concern, and does not combine the analysis results There is also no focus on cultural heterogeneity across regions.

R3: We thank your suggestion for our discussion. We have extended Section 6 and discussed the issues of concern in conjunction with the results from Section 5.

6 Discussion

In the long run of research, most of the Big Five personality traits are believed to be related to information sharing behavior on social media. The positive correlation between EXT traits and information sharing behavior is the highest (β = 0.05). The negative correlation between AGR traits and information sharing behavior is the greatest (β = -0.06). Fig,3-Fig.6 report significant correlations, therefore, H1, H2, and H4 were supported, H3 was rejected, and H5 was not fully supported. As shown in the above forest plots, literature [34] (n=21314) and literature [41] (n=409) have a relatively high weight, since the large sample size. Significantly, no study carries enough weight to decisively influence the outcome, suggesting that our meta-analysis has low sensitivity. Overall, our findings are shown in the Fig.10.

For cultural heterogeneity, we have already had a discussion—— “Cultural differences will affect the test results of the Big Five personality traits. For example, Europeans and Americans tend to have higher EXT scores compared to Asians and Africans. Unfortunately, inadequate research poses a hindrance to performing subgroup analysis.” In revised manuscript, we have further discussed this matter. Insufficient research can result in significant publication bias in meta-analysis. If the Leave-one-out method is used, the number of articles included may even be less than 5.

Cultural differences Cultural differences will affect the test results of the Big Five personality traits [65, 66]. For example, Europeans and Americans tend to have higher EXT scores compared to Asians and Africans. Unfortunately, inadequate research poses a hindrance to performing subgroup analysis. Insufficient research can result in significant publication bias in meta-analysis.
